# Linking Depressive and Anxiety Symptoms with Diet Quality of University Students: A Cross-Sectional Study during the COVID-19 Pandemic in India

**DOI:** 10.3390/healthcare10101848

**Published:** 2022-09-23

**Authors:** Satyajit Kundu, Najneen Rejwana, Md. Hasan Al Banna, Joseph Kawuki, Susmita Ghosh, Najim Z. Alshahrani, Natisha Dukhi, Subarna Kundu, Rakhi Dey, John Elvis Hagan, Christiana Naa Atsreh Nsiah-Asamoah, Suttur S. Malini

**Affiliations:** 1 School of Public Health, Southeast University, Nanjing 210009, China; 2 Faculty of Nutrition and Food Science, Patuakhali Science and Technology University, Patuakhali 8602, Bangladesh; 3 Department of Studies in Genetics and Genomics, University of Mysore, Mysuru 570005, India; 4 Department of Food Microbiology, Faculty of Nutrition and Food Science, Patuakhali Science and Technology University, Patuakhali 8602, Bangladesh; 5 Centre for Health Behaviours Research, Jockey Club School of Public Health and Primary Care, The Chinese University of Hong Kong, Hong Kong, China; 6 Department of Food Technology and Nutrition Science, Noakhali Science and Technology University, Noakhali 3814, Bangladesh; 7 Department of Nutrition Science, Purdue University, West Lafayett, IN 47907, USA; 8 Department of Family and Community Medicine, Faculty of Medicine, University of Jeddah, Jeddah 21589, Saudi Arabia; 9 Human Sciences Research Council, 116-118 Buitengracht Street, Cape Town 8001, South Africa; 10 Statistics Discipline, Khulna University, Khulna 9208, Bangladesh; 11 Department of Statistics, Government Brajalal College, National University of Bangladesh, Gazipur 1704, Bangladesh; 12 Department of Health, Physical Education & Recreation, College of Education Studies, University of Cape Coast, PMB TF0494, Cape Coast P.O. Box 5007, Ghana; 13 Neurocognition and Action-Biomechanics-Research Group, Faculty of Psychology and Sports Science, Bielefeld University, Postfach 10 01 31, 33501 Bielefeld, Germany; 14 Department of Clinical Nutrition and Dietetics, University of Cape Coast, PMB TF0494, Cape Coast P.O. Box 5007, Ghana

**Keywords:** anxiety, COVID-19, depression, diet quality, India, university students

## Abstract

This study examines the association of depressive and anxiety symptoms with diet quality among university students while controlling for different demographic and other health and lifestyle factors. This cross-sectional study was carried out between April 2021 and June 2021 among a total of 440 (unweighted) university students. Diet quality was assessed using a 10-item mini-dietary assessment index tool. The depressive and anxiety symptoms of participants were measured using the validated Patient Health Questionnaire-9 (PHQ-9), and the Generalized Anxiety Disorder (GAD-7) scale, respectively. Multivariable logistic regression and mediation analyses were performed. In this study, 61.1% (95% CI: 56.6% to 65.7%) of university students’ diet quality was good during the COVID-19 pandemic. Being a post-graduate student, an urban resident, having no depressive (AOR = 2.15, 95% CI: 1.20 to 3.84) and anxiety symptoms (AOR = 1.96, 95% CI: 1.07 to 3.59), no changes or improvement in appetite, and no changes in sleep duration were significantly associated with good diet quality among our study participants. Depressive and anxiety symptoms during COVID-19 had a significant effect on the diet quality of university students. Future public health policies need to be focused on improving the mental health and well-being of students particularly during pandemic situations to enhance their diet quality.

## 1. Introduction

After spreading to over 110 countries, Coronavirus disease (COVID-19) was declared a pandemic on 11 March 2020, by World Health Organization (WHO) [1,2,3]. To control the transmission of COVID-19, many international governments had to take rigorous decisions in form of lockdowns, travel restrictions and suspension of international flights, the closing of schools and business organizations, and bans on social gatherings, among others [4]. As a result, the most active age group, specifically the university students, got directly affected by these restrictive measures, which have a negative impact on their life both physically and mentally [4]. Moreover, these sudden changes also affected students’ learning environment, causing considerable uncertainties, as well as a halt in academic and career plans, thus adversely affecting their mental health [5]. 

Mental health problems among students are becoming a common problem, and about half of the university students have some minor mental health issues, including anxiety and depression [6]. These mental health problems have been reported to be associated with detrimental behaviours such as poor diet [7]. In addition, a healthy diet has been reported to be associated with behaviours like smoking, alcohol consumption, and physical activity among adolescents in East London [8]. Changes in appetite are one of the symptoms of depression, though other symptoms like reduced energy and a lack of interest in activities may influence one’s diet quality through lack of energy/motivation to prepare or enjoy meals. This, in turn, could influence the overall diet choices and thus diet quality [9]. Several studies show that healthier diets are associated with a lower risk of developing depression [9,10].

Recent evidence suggests that mental disorders are associated with poor diet quality [11,12], and in both developed and developing countries, depressive disorders are becoming significant health and economic burden [13]. Many shreds of evidence suggest that the onset of depression and anxiety is influenced by diet [14,15] and some modifiable lifestyle behaviours such as physical inactivity, smoking, etc. [13,16,17,18]. According to a recent meta-analysis of 24 separate cohorts with a combined total of 1,959,217 person-years, improved diet quality was linked to a decreased incidence of depressive symptoms regardless of the diet’s composition [10]. Several research studies on the relationship between dietary intake and sleep have found that short sleep duration and poor sleep quality are associated with an increase in unfavourable eating behaviours, such as poor diet quality and higher total energy consumption [19,20,21,22]. Another systematic review study argued that better sleep quality was linked to eating healthily, while consuming more processed meals high in free sugar was linked to poorer sleep quality [23].

Most of the previous literature [13,24,25] examined the effect of diet quality on depression and anxiety among children, women, and adults, but very few studies have focused on university students. To our knowledge, no study in India has explored the association of depression and anxiety with diet quality among university students. While there is existing information to suggest that individuals with poor sleep quality and psychological distress are likely to have an unhealthy dietary pattern, there is a dearth of information regarding the link between diet quality and depressive and anxiety symptoms among Indian university students. However, understanding this relationship is very crucial because university students go from late childhood to adulthood at a crucial transitional phase while making several significant life decisions that may cause different mental difficulties [26]. During this pandemic situation, the available studies have focused on the prevalence of mental health problems and their association with demographic and some lifestyle factors, but not diet quality. In our study, we tried to focus on university students’ diet quality during this COVID-19 pandemic and hypothesized that students with high depressive and anxiety symptoms might have poor diet quality. Thus, to address the current knowledge gaps and to better understand the relationship, this study investigated the association of depressive and anxiety symptoms with diet quality among university students in India during the COVID-19 pandemic, while controlling for the effects of specific demographic and other health and lifestyle factors.

## 2. Materials and Methods

### 2.1. Study Design and Participants

**Figure 1:**
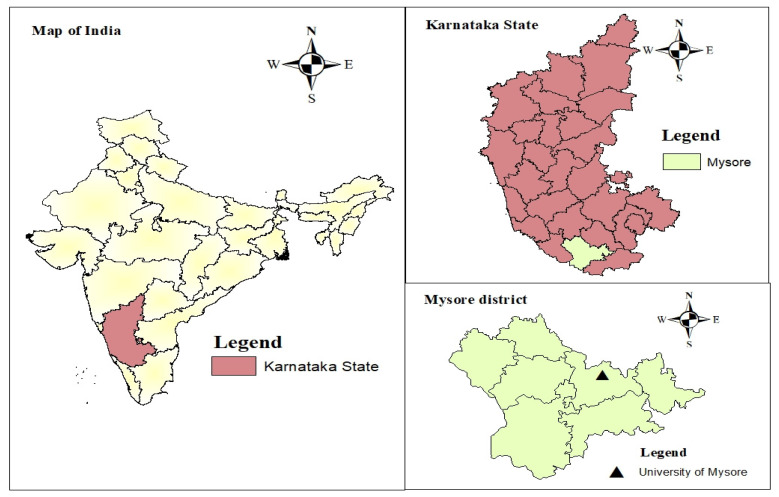
Geographical location of the study area.

It was a cross-sectional study that was carried out between April 2021 and June 2021 under the Helsinki Declaration ethical guidelines. This study was conducted among both undergraduate and postgraduate students from the University of Mysore (UoM) as well as from three affiliated colleges and centres of UoM. The UoM, a public state university, and its affiliated colleges are situated in Mysore city, Karnataka, India (see Figure 1).

### 2.2. Sampling and Study Procedure

**Figure 2:**
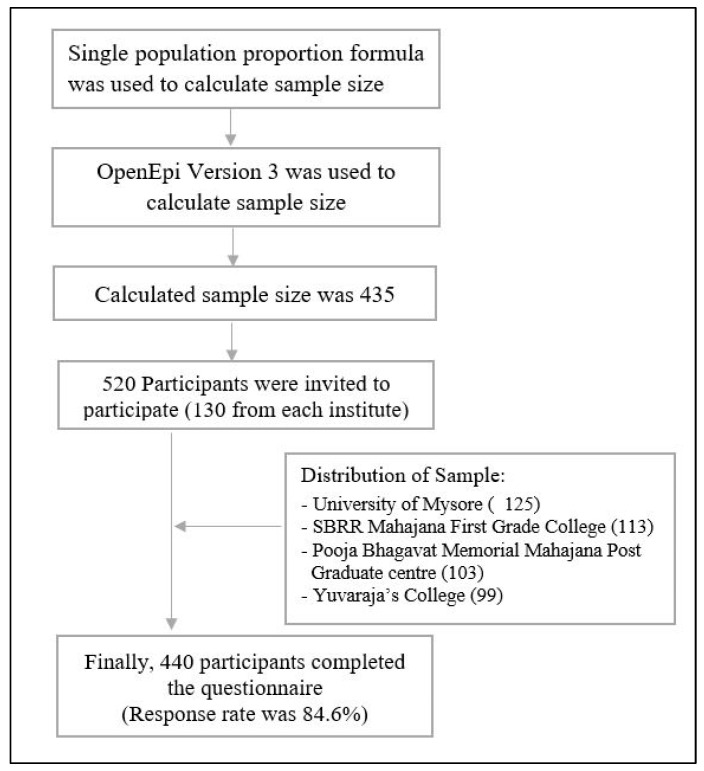
Flow chart of the sampling technique and participants selection.

The sample size was calculated using the single population proportion formula. Since there is no existing literature that assessed the diet quality of Indian university students, we considered the prevalence of depressive symptoms in a previous study [27] to calculate the sample size. We used OpenEpi Version 3 (https://www.openepi.com/SampleSize; accessed on 3 April 2021) with the assumption of 95% confidence level, 5% absolute precision, a design effect of 1.2, and taking 38% prevalence of depressive symptoms among Indian university students [27]. After calculating, our final desired sample size was 435. We used a simple random sampling technique to select the participants. All the undergraduate and postgraduate students from UoM and its three affiliated colleges who were willing to participate were included. Participants who had any physical difficulties and disabilities as well as any chronic diseases were excluded. All the participants were interviewed through a face-to-face approach by trained research staff using a structured questionnaire. The questionnaire was piloted among a sample of 30 university students who were recruited randomly. Preliminarily, we invited approximately 500 university students face-to-face to attain our desired sample. We distributed the sample as around 130 participants from each of the 4 institutes (UoM and its three affiliated colleges and centres) in order to attain the desired sample size. Finally, a total of 440 participants agreed to participate voluntarily and took part in the interview (125 participants from UoM, 113 participants from SBRR Mahajana First Grade College, 103 participants from Pooja Bhagavat Memorial Mahajana Post Graduate centre, 99 participants from Yuvaraja’s College). The details of the sampling and sample size have been shown in Figure 2. The participants were informed that anonymity and confidentiality would be maintained, and the data would be used only for research purposes. Written informed consent was also obtained from all participants. Only participants who were willing to participate and filled out written consent were interviewed, making the final response rate of about 88.0%.

### 2.3. Measures

#### 2.3.1. Diet Quality

A 10-item mini-dietary assessment index tool was used to assess the diet quality of study participants [28]. The mini-dietary assessment tool was used to evaluate overall diet quality based on the Dietary Guidelines for Indians by the National Institute of Nutrition [29]. The index comprised four food groups that should be consumed, four food groups that should be consumed at a limited amount, and the other two food groups regarding meal skipping and regular diet. Responses to food groups of which adequate amounts should be consumed were reported using a 5-point Likert scale where “1” was for seldom, “3” was for sometimes, and “5” was for always. Responses to food groups that should be consumed in limited quantities were also reported using a 5-point Likert scale where “1” was for always, “3” was for sometimes, and “5” was for seldom. The maximum possible score for diet quality using this assessment tool was 50. A participant’s diet quality was considered “good” when his/her total score was greater than or equal to 30, and if the score was below 30, then the diet quality was defined as “poor” [30]. In the present study, the internal consistency of the assessment tool was adequate (Cronbach’s alpha = 0.82).

#### 2.3.2. Depressive Symptoms

Patient Health Questionnaire (PHQ-9), a 9-item scale, was used to assess the level of depressive symptoms. The PHQ-9 is a validated tool for measuring depressive symptoms [31], and has also been used in several previous studies among university students [32,33,34]. The PHQ-9 scale is composed of 9 individual questions, where the response for each question was coded based on a four-point Likert scale ranging from “Not at all” (0) to “Nearly every day” (3). The total score ranges from 0 to 27, where a higher score indicates more severe depressive symptoms. After summarizing the total score obtained from the responses of the nine items, the summed score was categorized into two groups. Participants with a score of <10 were classified as having no depressive symptoms, and those who made a score of 10 or more were categorized as having depressive symptoms [32,35]. The reliability of the scale in the present study was high (Cronbach’s α = 0.83).

#### 2.3.3. Anxiety Symptoms

The Generalized Anxiety Disorder (GAD-7) tool, a 7-item scale, was used to assess the anxiety symptoms level of study participants. The GAD-7 is a valid and reliable tool for screening anxiety symptoms in epidemiological studies [33,34]. The GAD-7 scale includes seven items anchored on a four-point Likert scale ranging from 0 (Never) to 3 (Nearly every day). The total score ranges from 0 to 21, where a higher score indicates more severe anxiety symptoms. In this study, those scoring ≥10 were defined as having the existence of anxiety symptoms [33,36]. The reliability of the scale in the present study was sufficient (Cronbach’s α = 0.79).

#### 2.3.4. Other Health and Lifestyle Characteristics

The level of physical activity (PA) during the COVID-19 lockdown was assessed using a fixed-choice question: ‘‘on the average every day how much time was spent actively’’, where the respondents chose one of three following categories describing their activity time: (1) Low PA (<0.5 h a day); (2) Moderate PA (0.5–2 h a day), and (3) High PA (>2 h a day) [37]. Changes in physical activity level during lockdown were assessed using a single fixed-choice question: ‘‘During the COVID-19 lockdown, my average physical activity level a day was: (1) Decreased, (2) Not changed, (3) Increased’’ [37]. We estimated the total sleep duration a day of study participants by asking the following question: “How many hours a night on average did you sleep during the past 4 weeks?” We categorized sleep duration into long (more than 8 h), normal (7 and 8 h), and short (6 h or less) [32,33,38]. Changes in average sleep duration a day were assessed using a single fixed-choice question: ‘‘During the COVID-19 lockdown, my average sleep duration a day was: (1) Decreased, (2) Not changed, (3) Increased’’. Changes in meal size during the COVID-19 lockdown were assessed by asking a close-ended question: ‘‘During the COVID-19 lockdown, on average my meal size was: (1) Decreased, (2) Not changed, (3) Increased’’. Similarly, the changes in appetite were also measured using a fixed-choice question: ‘‘During the COVID-19 lockdown, on average my appetite was: (1) Worse than before, (2) Poorer than before, (3) Not changed, (4) Better than before, and (5) Do not know’’.

### 2.4. Data Analysis

Frequencies and percentages were used to interpret the descriptive characteristics of study participants. A chi-square test was performed to identify the association between outcome and independent variables. In the univariable analyses, the effect of the independent variables on the diet quality of university students was estimated by simple logistic regression analysis (Block-0). Multivariable logistic regression models were also employed using three Blocks. In Block-1 (demographic characteristics), we included sex, age, education level, study course, residence, and living situation during the lockdown. In Block-2 (health and lifestyle characteristics), we included smoking status, physical activity level and change during the lockdown, average sleep time a day and its changes during the lockdown, alcohol use, changes in meal size, and appetite. In Block-3, all the demographic variables, living situation, health and lifestyle characteristics, and their changes during the COVID-19 lockdown were included. The crude and adjusted odds ratios are demonstrated with 95% confidence interval (95% CI). Multicollinearity among variables for each model was checked using variance inflation factors (VIF) and tolerance values. For data analysis, we used the statistical package STATA (version 16.0).

## 3. Results

### 3.1. Distribution of Diet Quality across Different Socio-Demographic Characteristics of Study Participants during the COVID-19 Pandemic in India

A total of 440 university students were included in this study. Of the total participants, the majority were females (54.8%), aged between 18 and 25 years (96.4%), postgraduate students (63.2%), and studying business and law courses (52.3%). Moreover, the majority of the students resided in semi-urban and urban areas (71.4%) and lived with their families (80.5%). In the present study, we found that 61.1% (95% confidence interval [95% CI]: 56.6% to 65.7%) of university students had good diet quality, as shown in Table 1.

About 66% of male students had good diet quality compared to 56% of female students (*p* < 0.05), while 65% of students with science/engineering background had a good quality diet compared to 48% of students of arts/social science subjects (*p* < 0.05). A total of 66% of students from urban areas had a good quality diet, while half of the students from rural areas had a poor diet quality (*p* < 0.05), as shown in Table 1.

### 3.2. Health and Lifestyle Characteristics

Regarding health and lifestyle characteristics, the majority of the respondents were non-smokers (88.2%) and never drink alcohol (73.2%). More than half of the respondents were engaged in moderate physical activity (58.2%) and 51.4% reported a change in their physical activity during the COVID-19 lockdown. Almost half of the participants (49.1%) reported that their average sleep duration was long (>8 h a day). About 30.2% of students reported that their meal sizes decreased during the lockdown period. In the present study, 26.8% of respondents had depressive symptoms, while 22.9% of participants had anxiety symptoms (Table 2).

In case of changes in sleep duration, the highest percentage (69.3%) of having good diet quality was among those who did not experience any changes in their sleep duration compared to those whose sleep duration was decreased (*p* < 0.05). A similar pattern was also observed for changes in meal size, where those who experienced a decreased meal size during COVID-19 had poor diet quality compared to those who experienced no changes in their meal size. Participants having depressive (55.9%) and anxiety symptoms (55.5%) had a poor diet quality compared to those with no depressive (32.6%) and anxiety (33.9%) symptoms (*p* < 0.05), as shown in Table 2.

### 3.3. Logistic Regression Analysis on Socio-Demographic, Health, and Lifestyle Factors on Diet Quality

Table 3 represents the detailed results of four different blocks of logistic regression. The unadjusted model has been shown in Block-0, while only the socio-demographic factors were adjusted in Block-I. The variables related to health and lifestyle and their changes during the COVID-19 lockdown were adjusted in Block-2. These were just to present the variables that were significant in Block-1 and Block-2 and to estimate how those variables are modified to each other in the final model (Block-3; Table 3).

In the final model (Block-3), all the variables (socio-demographic, health, and lifestyle characteristics) were included at the same time. After adjusting all the variables, education level, study course, residence, change in sleep duration, change in appetite, depressive symptoms, and anxiety symptoms were found to be significant factors associated with good diet quality. Participants from arts/social science and other courses were 35% (AOR = 0.35, 95% CI: 0.16 to 0.81) and 18% (AOR = 0.18, 95% CI: 0.07 to 0.49), respectively, and were found to be less likely to have good diet quality compared to their counterparts from science/engineering courses. Compared to participants residing in rural areas, those in urban areas were 93% more likely to have good diet quality (AOR = 1.93, 95% CI: 1.13 to 3.31), while those with no change in their sleep duration were 2.45 times more likely to have good diet quality compared to those with decreased sleep duration (AOR = 2.45, 95% CI: 1.26 to 4.77). Similarly, participants with no change in their appetite and those with better-than-before appetite were 7.53 times (AOR = 7.53, 95% CI: 2.56 to 16.34) and 12.98 times (AOR = 12.98, 95% CI: 5.63 to 18.0), respectively, more likely to have good diet quality compared to those with worse appetite than before. Lastly, participants with no depressive (AOR = 2.15, 95% CI: 1.20 to 3.84) and anxiety symptoms (AOR = 1.96, 95% CI: 1.07 to 3.59) had 2.15 times and 1.96 times higher odds of having good diet quality compared to those who had depressive and anxiety symptoms, respectively (Table 3).

## 4. Discussion

Due to COVID 19 mental health has been impacted globally among all people. Eating behaviours like over and under-eating and others are being triggered by anxiety, and depression [39]. Our study aimed to identify the association of depressive and anxiety symptoms with diet quality.

The finding of this study indicates that arts and social science course students are less likely to have good diet quality than science/engineering course students. This might be due to theoretical lessons being replaced by written documents and multimedia classes as compared to science/engineering which faced difficulty in terms of practical sessions that were predominantly lab-based and required hands-on learning [40,41]. Again, it is likely that students enrolled in science-related programmes have some insight or considerable knowledge of nutrition and its role in supporting optimal health as compared to their counterparts studying arts-related courses. This finding is supported by that of a previous study among university students in Puerto Rico where it was found that eating habits were associated with knowledge of nutrition and students’ academic programme of study [34]. Evidence from previous studies [42,43] indicated that students from the art courses had a substantial tendency to develop mental illnesses as compared to students studying engineering and business.

Students in urban areas were more likely to have good diet quality compared to those from rural areas. An explanation could be that students in urban areas have greater access to food and digital platforms as compared to their rural counterparts [44,45]. Hence, online food ordering could not easily be possible for students from rural areas. This highlights the major differences in digital equipment availability between students from urban and rural areas, due to the very quick transition to virtual learning, which did not allow students without experience to have the confidence and assistance to gain dietary knowledge [44,46]. Another explanation is that usually urban people are more likely to be in the higher socio-economic class as compared to those residents in rural communities. It may be possible that students who belonged to a high socioeconomic class were financially empowered and therefore at an advantage of consuming high-quality meals as compared with rural people.

Regarding sleep duration, our study is consistent with the literature that indicates sleep patterns being maintained are associated with food lifestyle [47,48]. Additionally, previous studies also identified that better sleep quality resulted in better eating habits based on experimental investigations highlighting the links between eating habits and sleep quality [49,50]. The sleep-wake cycle and quality of sleep are related to meal timing. If meals are eaten at regular intervals, with correct food choices, it is more likely to impact the quality of sleep positively as metabolic and physiological processes respond accordingly based on the circadian rhythm [51,52]. In addition, those spending more time at home are more likely to make informed food choices, have adequate time for cooking, and do not have to skip meals [48,53].

We found a significant association between mental health (i.e., depressive and anxiety symptoms) and the diet quality of university students. The association between mental health and diet is well-established [54]. Lower depressive and anxiety symptoms have been linked to healthier diets. The link between mental health and dietary intake is consistent in various studies [55,56,57], with lower healthy food intake indicating an increase in anxiety and depression. In contrast, poor diets have been closely associated with greater risks of anxiety and depression [58,59]. Depressed individuals are more likely to consume a greater amount of calories, have poorer quality of diet, and have a higher body mass index (BMI) [60]. Those with greater depression and anxiety levels tend to not follow careful food selection and eat larger quantities and more than they need to, thus regulating emotions via food [61,62]. Similarly, a previous study also found a significant association between anxiety disorders and lower diet quality, where people with an anxiety disorder had a lower healthy eating index compared to those having no anxiety disorders [63]. Admittedly, depending on the symptoms that arise due to the anxiety disorder(s), a person’s behaviour may become altered [63]. Another longitudinal research that measured participants’ adherence to the Mediterranean diet found, as an additional outcome, that there were statistically significant connections between participants’ present anxiety disorders and their likelihood of having a lower quality diet [9]. Food intake can impact the regulation of emotions and mood, thereby affecting food choices, resulting in a bidirectional influence on diet quality. A mutual connection exists between poor diet and low mood, and vice versa. The quality of the diet can be impacted by different psychological states or stressful conditions [15].

The finding that students with a decreased sleep duration were more likely to have a reduction in their diet quality is consistent with previous studies [54,55,56,57]. It has been shown that when students stay awake late into the night (likely studying or engaging in social media activities), they are likely to nibble on unhealthy fatty and carbohydrate-based snacks such as sugar-sweetened beverages, candies, crisps, and chocolate, which reduces their diet quality. Comparatively, other studies have also revealed that poor dietary intake and unhealthy eating habits during the day can significantly reduce one’s quality of sleep or result in sleep deprivation at night [58,59,60,61].

### Strengths and Limitations

This is one of the first studies that has attempted to identify the association between depressive and anxiety symptoms and the diet quality of university students in India. The results of this study offer policymakers and public health professionals a starting point resource (i.e., baseline information) to help with the design and implementation of evidence-based interventions and efforts to enhance diet quality among Indian university students. The first limitation of this study is its cross-sectional nature in design; thus, any causal relationship cannot be identified, and the study was conducted at one academic institution and its affiliated colleges. Hence, a longitudinal study can investigate the long-term impact of the pandemic on anxiety and depression with diet quality. To extend and generalize the findings, large-scale surveys should be conducted in various universities in India. All data were self-reported, and a systematic clinical evaluation would provide a better assessment of anxiety and depression. Self-reporting may include social desirability and recall bias, whereby under-and overestimation occurred regarding participant perception of some lifestyle aspects during the COVID-19 pandemic.

## 5. Conclusions

The study results indicated that changes in sleep duration, appetite, residence, study course, education level, depressive symptoms, and anxiety symptoms were significantly associated with diet quality. To increase the quality of diet in Indian university students, consideration should be given to the associated factors identified in this study. Since psychological factors play a significant role in having good diet quality, it is imperative that intervention strategies could be developed and implemented for the promotion of mental health during the pandemic so that diet quality can be maintained. There is also a need for the provision of mental health support services during times of confinement so that good diet quality can be maintained. In addition, a large-scale follow-up study including many other clinical factors that are associated with dietary choices could also be conducted among Indian university students to ascertain the evidence for intervention design.

## Figures and Tables

**Table 1:** Distribution of diet quality across different socio-demographic characteristics of study participants during the COVID-19 pandemic in India (n = 440).

Characteristics	Categories	Total; n (%)	Diet Quality		*p* Values
			Poor; n (%)	Good; n (%)	
Sex	Male	199 (45.2)	67 (33.7)	132 (66.3)	**0.042**
	Female	241 (54.8)	104 (43.2)	137 (56.9)	
Age in years	18–21	228 (51.8)	100 (43.7)	128 (56.1)	**0.037** *
	22–25	196 (44.6)	68 (34.7)	128 (65.3)	
	>25	16 (3.6)	3 (18.8)	13 (81.2)	
Education level	Under-graduate	162 (36.8)	60 (37.0)	102 (63.0)	0.548
	Post-graduate	278 (63.2)	96 (34.5)	182 (65.5)	
Study course	Science/engineering	115 (26.1)	40 (34.8)	75 (65.2)	**0.009**
	Arts/social science	68 (15.5)	35 (51.5)	33 (48.5)	
	Business and law	230 (52.3)	80 (34.8)	150 (65.2)	
	Others ^#^	27 (6.1)	16 (59.3)	11 (40.7)	
Residence	Rural	126 (28.6)	63 (50.0)	63 (50.0)	**0.010**
	Semi-urban	87 (19.8)	31 (35.6)	56 (64.4)	
	Urban	227 (51.6)	77 (33.9)	150 (66.1)	
Living situation during lockdown	Alone	21 (4.8)	8 (38.1)	13 (61.9)	0.970
	With partner	27 (6.1)	11 (40.7)	16 (59.3)	
	With family	354 (80.5)	136 (38.4)	218 (61.6)	
	With friends/others	38 (8.6)	16 (42.1)	22 (57.9)	
Diet quality	Poor	171 (38.9)	-	-	-
	Good	269 (61.1)	-	-	

^#^ Others included humanity and education. * *p*-value was obtained from Fisher’s exact test. Bolded *p* values indicate statistical significance.

**Table 2:** Health and lifestyle characteristics and their changes during the COVID-19 lockdown of study participants (n = 440).

Characteristics	Categories	Total; n (%)	Diet Quality		*p* Values
			Poor; n (%)	Good; n (%)	
Smoking status	More than before	21 (4.8)	7 (33.3)	14 (66.7)	0.926 *
	Less than before	15 (3.4)	7 (46.7)	8 (53.3)	
	Same as before	7 (1.6)	3 (42.9)	4 (57.1)	
	Quit smoking	9 (2.1)	4 (44.4)	5 (55.6)	
	Do not smoke	388 (88.2)	150 (38.7)	238 (61.3)	
Level of physical activity	Low (0.5 h/day)	126 (28.6)	53 (42.1)	73 (57.9)	0.641
	Moderate (0.5–2 h/day)	256 (58.2)	95 (37.1)	161 (62.9)	
	High (>2 h/day)	58 (13.2)	23 (39.7)	35 (60.3)	
Changes in physical activity	Decreased	109 (24.8)	39 (35.8)	70 (64.2)	0.739
	No change	214 (48.6)	86 (40.2)	128 (59.8)	
	Increased	117 (26.6)	46 (39.3)	71 (60.7)	
Sleep duration	Short (<6 h/day)	43 (9.8)	16 (37.2)	27 (62.8)	0.277
	Normal (6–8 h/day)	181 (41.1)	63 (34.8)	118 (65.2)	
	Long (>8 h/day)	216 (49.1)	92 (42.6)	124 (57.4)	
Changes in sleep duration	Decreased	95 (21.6)	45 (47.4)	50 (52.6)	**0.023**
	No change	150 (34.1)	46 (30.7)	104 (69.3)	
	Increased	195 (44.3)	80 (41.0)	115 (59.0)	
Alcohol use	Never	322 (73.2)	131 (40.7)	191 (59.3)	0.433
	Monthly or less	68 (15.5)	23 (33.8)	45 (66.2)	
	≥2 times a month	50 (11.4)	17 (34.0)	33 (66.0)	
Meal size	Decreased	133 (30.2)	60 (45.1)	73 (54.9)	**0.017**
	No change	210 (47.7)	67 (31.9)	143 (68.1)	
	Increased	97 (22.1)	44 (45.4)	53 (54.6)	
Appetite	Worse than before	14 (3.2)	11 (78.6)	3 (21.4)	**0.000**
	Poorer than before	88 (20.0)	47 (53.4)	41 (46.6)	
	No change	204 (46.4)	66 (32.4)	138 (67.6)	
	Better than before	90 (20.5)	25 (27.8)	65 (72.2)	
	Do not know	44 (10.0)	22 (50.0)	22 (50.0)	
Depressive symptoms	Yes	118 (26.8)	66 (55.9)	52 (44.1)	**0.000**
	No	322 (73.2)	105 (32.6)	217 (67.4)	
Anxiety symptoms	Yes	101 (22.9)	56 (55.5)	45 (44.5)	**0.000**
	No	339 (77.1)	115 (33.9)	224 (66.1)	

* *p*-value was obtained from Fisher’s exact test. Bolded *p* values indicate statistical significance.

**Table 3:** Different models of regression analysis show the factors associated with study participants’ good diet quality.

Variables	Block-0	Block-1	Block-2	Block-3
	COR (95% CI)	AOR (95% CI)	AOR (95% CI)	AOR (95% CI)
**Socio-demographic characteristics**				
**Sex**				
Male	**1.50** * (1.01, 2.21)	1.29 (0.83, 1.99)		1.21 (0.72, 2.04)
Female	Ref	Ref		Ref
**Age in years**				
18–21	Ref	Ref		Ref
22–25	1.47 (0.99, 2.18)	1.52 (0.97, 2.41)		1.72 (1.03, 2.87)
>25	3.39 (0.94, 12.20)	3.28 (0.85, 12.68)		3.59 (0.80, 16.09)
**Education level**				
Under-graduate	Ref	Ref		Ref
Post-graduate	1.89 (1.59, 2.32)	1.62 (1.38, 2.01)		**1.42** ** (1.24, 2.73)
**Study course**				
Science/engineering	Ref	Ref		Ref
Arts/social science	**0.50** * (0.27, 0.93)	0.51 (0.26, 1.02)		**0.35** * (0.16, 0.81)
Business and law	1.0 (0.63, 1.60)	0.89 (0.52, 1.50)		0.92 (0.51, 1.68)
Others ^#^	**0.37** * (0.16, 0.86)	**0.29** ** (0.12, 0.71)		**0.18** ** (0.07, 0.49)
**Residence**				
Rural	Ref	Ref		Ref
Semi-urban	**1.81** * (1.03, 3.16)	1.52 (0.83, 2.78)		1.42 (0.72, 2.82)
Urban	**1.95** ** (1.25, 3.04)	**1.74** * (1.08, 2.80)		**1.93** * (1.13, 3.31)
**Living situation**				
Alone	Ref	Ref		Ref
With partner	0.90 (0.28, 2.88)	1.48 (0.42, 5.13)		1.63 (0.39, 6.84)
With family	0.99 (0.40, 2.44)	1.35 (0.51, 3.56)		1.27 (0.41, 3.92)
With friends/others	0.85 (0.28, 2.52)	0.84 (0.27, 2.62)		1.47 (0.39, 5.60)
**Health and lifestyle characteristics and their changes during the COVID-19 lockdown**				
**Smoking status**				
More than before	Ref		Ref	Ref
Less than before	0.57 (0.15, 2.23)		0.46 (0.10, 2.10)	0.36 (0.07, 1.81)
Same as before	0.67 (0.12, 3.84)		0.60 (0.09, 4.02)	0.39 (0.05, 2.72)
Quit smoking	0.63 (0.13, 3.09)		0.66 (0.11, 3.81)	0.48 (0.08, 3.01)
Do not smoke	0.79 (0.31, 2.01)		0.99 (0.36, 2.80)	0.90 (0.29, 2.76)
**Level of physical activity**				
Low (0.5 h/day)	Ref		Ref	Ref
Moderate (0.5–2 h/day)	1.23 (0.80, 1.90)		0.90 (0.54, 1.48)	1.19 (0.69, 2.06)
High (>2 h/day)	1.10 (0.59, 2.08)		1.04 (0.50, 2.15)	1.22 (0.56, 2.66)
**Changes in physical activity**				
Decreased	Ref		Ref	Ref
No change	0.83 (0.51, 1.34)		0.84 (0.49, 1.43)	0.95 (0.54, 1.68)
Increased	0.86 (0.50, 1.47)		0.69 (0.37, 1.27)	0.67 (0.35, 1.29)
**Sleep duration**				
Short (<6 h/day)	Ref		Ref	Ref
Normal (6–8 h/day)	1.11 (0.56, 2.21)		0.99 (0.46, 2.15)	1.29 (0.56, 2.96)
Long (>8 h/day)	0.80 (0.41, 1.57)		0.83 (0.38, 1.82)	1.14 (0.49, 2.68)
**Changes in sleep duration**				
Decreased	Ref		Ref	Ref
No change	**2.03** ** (1.20. 3.46)		**1.96** * (1.06, 3.64)	**2.45** ** (1.26, 4.77)
Increased	1.29 (0.79, 2.12)		1.39 (0.77, 2.50)	1.61 (0.86, 3.00)
**Alcohol use**				
Never	Ref		Ref	Ref
Monthly or less	1.34 (0.77, 2.32)		1.39 (0.75, 2.56)	1.03 (0.52, 2.03)
≥2 times a month	1.33 (0.71, 2.49)		1.34 (0.64, 2.81)	0.82 (0.37. 1.83)
**Meal size**				
Decreased	Ref		Ref	Ref
No change	**1.75** * (1.12, 2.75)		0.85 (0.47, 1.56)	0.78 (0.41, 1.49)
Increased	0.99 (0.59, 1.67)		0.67 (0.35, 1.28)	0.59 (0.29, 1.17)
**Appetite**				
Worse than before	Ref		Ref	Ref
Poorer than before	3.20 (0.83, 12.26)		2.88 (0.67, 12.36)	3.73 (0.78, 17.74)
No change	**7.67** ** (2.07, 10.41)		**6.44** * (2.47, 11.18)	**7.53** * (2.56, 16.34)
Better than before	**9.53** ** (5.45, 17.05)		**9.73** ** (5.18, 17.47)	**12.98** ** (5.63, 18.0)
Do not know	3.67 (0.90, 14.97)		3.18 (0.68, 14.90)	2.91 (0.57, 14.72)
**Depressive symptoms**				
Yes	Ref		Ref	Ref
No	**2.62** *** (1.70, 4.04)		**1.82** * (1.07, 3.10)	**2.15** ** (1.20, 3.84)
**Anxiety symptoms**				
Yes	Ref		Ref	Ref
No	**2.42** *** (1.54, 3.81)		**1.79** * (1.02, 3.12)	**1.96** * (1.07, 3.59)
**Goodness-of-fit test**				
LR chi^2^ (*p* value)		29.71 (0.003)	57.98 (0.000)	95.72 (0.000)
Hosmer-Lemeshow chi^2^ (*p*-value)		3.30 (0.914)	7.41 (0.116)	13.53 (0.095)

* *p* < 0.05; ** *p* < 0.01; *** *p* < 0.001; ^#^ Others included humanity and education. Bolded values indicate statistical significance. Note. COR = crude odds ratio; AOR = adjusted odds ratio; CI = confidence interval.

## Data Availability

The study data are available upon request from the corresponding author.
